# Increasing Physical Activity in Educational Settings Using Mixed Reality Technology: Iterative Formative Study

**DOI:** 10.2196/83556

**Published:** 2026-03-25

**Authors:** Katherine E Spring, Calvin W Feldt, Matthew Parker Posey, Phillip Nauta, Senlin Chen, David C Shepherd, Amanda E Staiano

**Affiliations:** 1 Pennington Biomedical Research Center, 6400 Perkins Rd., Baton Rouge, LA, 70808, United States, 1 2257632729; 2 Division of Computer Science and Engineering, Louisiana State University, Baton Rouge, LA, United States; 3 School of Kinesiology, Louisiana State University, Baton Rouge, LA, United States

**Keywords:** digital learning tools, middle school, mixed reality, school-based physical activity, active learning technologies

## Abstract

**Background:**

As physical education classes are lost to budget cuts and recess is canceled to meet standardized testing goals, the modern school day has become dominated by sedentary digital activities. To reverse this trend, current interventions have focused on reducing screen time. However, instead of fighting this digital invasion, this study examined the use of technology, specifically mixed reality, to turn screen time from sedentary into active time, promoting physical activity in a classroom setting.

**Objective:**

The primary aim of this study was to iteratively develop and test a mixed reality prototype that promotes physical activity (eg, jumping, squatting, and punching) during a digital classroom activity. The primary outcomes were the percentage of active time during the activity, a breakdown of the intensity of that active time, and an evaluation of the prototype’s usability.

**Methods:**

Between November 2023 and April 2025, a multidisciplinary research team developed a prototype and evaluated it during 2 rounds of pilot-testing. Participants were aged 10 to 15 years and attended local middle schools. Physical activity was assessed using a medical-grade, hip-worn accelerometer (ActiGraph wGT3X-BT). Acceptability was assessed using a validated questionnaire (the System Usability Scale) that has a maximum score of 100. To collect feedback for prototype improvements, semistructured interviews were conducted after each round of pilot-testing.

**Results:**

In the first round of pilot-testing, students (n=22) were active for 46.0% (6.9, SD 2.7 minutes) of the headset session, which lasted 15 (SD 0) minutes. After improving the prototype using feedback from the first round, students in the second round (n=10) were active for 5.8 (SD 3.1) minutes (62.4%) of the web-based assignment, which lasted 9.3 (SD 2.41) minutes, while still reporting “good” acceptability scores (mean 73.8, SD 17.2). There were no significant differences in acceptability ratings between the 2 pilot-testing rounds (*P*=.16), nor were there differences between boys and girls in round 1 (*P*=.79) or round 2 (*P*=.61).

**Conclusions:**

The results of this iterative study indicate that mixed reality can be used to elicit physical activity in a classroom setting, at least for short assignments. However, further research is needed to determine longer-term use and effectiveness.

## Introduction

Students in the United States spend close to 7 hours each weekday in school [1], and for most of that time, they are sedentary [2]. When students transition from elementary to secondary school, academic pressures increase, and there tends to be an increase in sedentary activity [3]. In total, 89% of middle school students report being physically active for less than the recommended 60 minutes per day [4]. While recent educational policy is aimed at increasing students’ academic performance [5,6], the policies have unintentionally led to decreased opportunities for physical activity [7] and increased use of sedentary digital learning tools [8].

The use of digital learning tools (ie, tablets, laptop computers, and smart boards) in school settings is not a new concept [9], but the COVID-19 pandemic catalyzed rapid adoption [10] as many schools switched to remote learning, contributing to significant increases in screen time for children [11,12]. Upon returning to in-person learning, the incorporation of technology into the curriculum became pervasive [13,14], further challenging educators and parents to find ways to decrease sedentary behaviors. Previous school-based interventions have focused on strategies such as physical activity breaks, active lessons, teacher training, school policy to limit screen time, and classroom modifications to encourage movement [15]. Most of these interventions focus on moderate to vigorous physical activity yet neglect light physical activity, a lower intensity of movement that is related to positive health [16,17] and academic outcomes [17,18] and may be more feasible within the space limitations of an indoor classroom. Additionally, many school-based physical activity interventions avoid technology altogether. The development and deployment of new technology, such as mixed reality, could serve as a novel approach to leverage technology as a tool to increase physical activity in the school classroom.

Mixed reality devices, most commonly headsets, are equipped with cameras that allow users to view the real world around them. This allows them to operate headsets safely without fear of collision, which was a common problem with virtual reality headsets. Furthermore, these headsets now support technology that allows for identification and interpretation of gross motor movements of the arms, hands, and head, allowing for controller-free, direct interaction between user and application [19]. A recent systematic review reported that there is preliminary evidence suggesting that mixed reality headsets could be used to increase physical activity through exergaming [20]. More specifically, 2 popular virtual reality exergames, Gorilla Tag and Beat Saber, have been shown to increase light to moderate physical activity in early adolescents [21]. Some researchers have started investigating the use of mixed reality headsets in physical education classes [22], but it is currently unknown whether mixed reality headsets could be incorporated into traditional school curriculums, replacing sedentary computer time while encouraging more active learning environments.

The purpose of this study was to design a mixed reality prototype using an iterative process to optimize physical activity and satisfaction. The population of interest was middle school students, and the prototype was designed to be integrated into typical online academic assignments (ie, reading comprehension and vocabulary). The prototype program required students to punch, jump, and squat to complete the online academic tasks. The primary aim of this study was to determine how active students were when using the mixed reality technology to complete online academic assignments. The secondary aim was to examine the students’ acceptability of the mixed reality prototype.

## Methods

### Study Design and Setting

**Figure 1.**
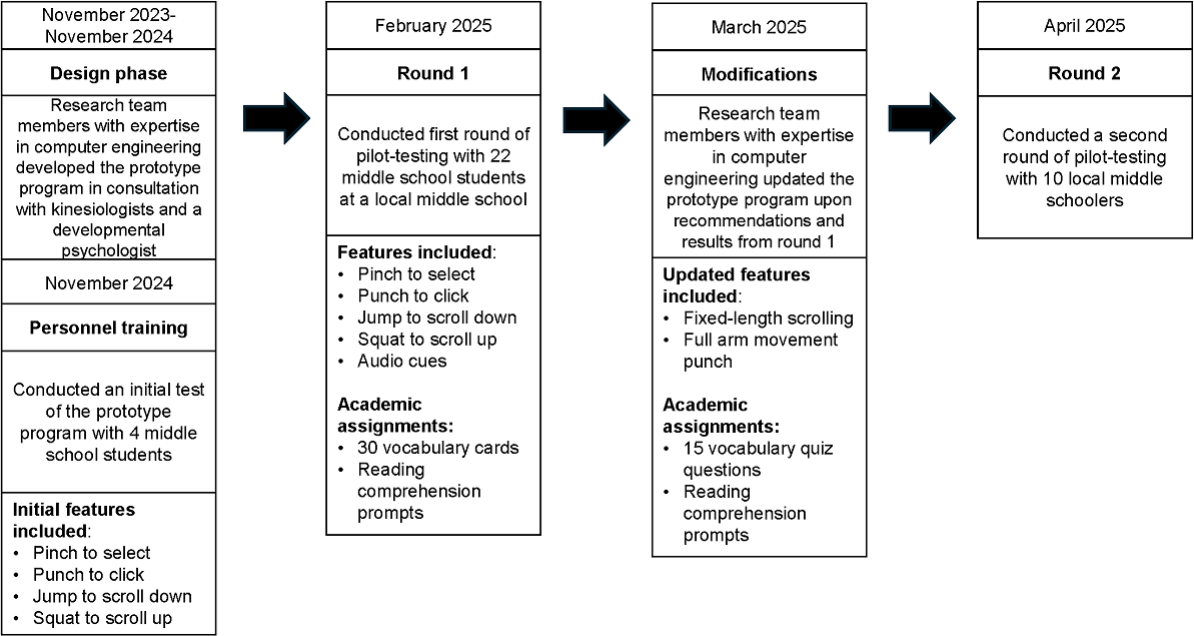
Iterative pilot study timeline of participant recruitment, prototype features, and modifications.

This iterative study consisted of four phases: (1) prototype design and personnel training, (2) round 1 pilot-testing, (3) prototype modifications, and (4) round 2 pilot-testing. A detailed timeline can be found in Figure 1. The study was led by a multidisciplinary team trained in computer science, kinesiology, and developmental psychology. Prior to data collection, the research team determined a priori the study end points (metrics of success) that would inform the number of iterative testing and modifications. Specifically, the prototype would be considered complete once the product (1) engaged users in physical activity (ranging from light to moderate to vigorous) for at least 50% of the time spent completing an online academic assignment on average and (2) achieved an average score of at least 60 on the System Usability Scale (SUS) [23,24]. While developing the prototype, the research team had several discussions regarding the movements that the prototype would use, with the limitations of the Meta Quest 3 (Reality Labs) in mind. The team aimed to encourage movement of the upper and lower body while being mindful to minimize repetitive movements as they could cause injury. The Meta Quest 3 does not support feet tracking, but it does support hand and head tracking. On the basis of these constraints, the research team determined that punching would be used to elicit upper-body movements, enabled via hand tracking, and squatting and jumping would elicit lower-body movements, enabled via head tracking.

From November 2023 to November 2024, members of the research team designed a mixed reality prototype program that, when paired with a Meta Quest 3 headset, would allow students to use their body as a “mouse” to complete an online academic assignment. Personnel training took place in November 2024. During this time, 4 middle school students used the prototype to allow the research team to refine the study protocol and prototype program. Informal conversations with the students indicated that the prototype was enjoyable and worked as expected but could benefit from the addition of audio cues. This feedback was incorporated into the prototype prior to pilot-testing. Round 1 of pilot-testing took place in February 2025 at a local middle school. On the basis of the results of round 1, minor modifications were made to the prototype in March 2025. In April 2025, round 2 of pilot-testing took place at a laboratory space on the Louisiana State University campus.

### Participants and Procedures

To be eligible to participate in the pilot-testing of the prototype, students had to be aged between 10 and 15 years. Students were excluded if they did not have parental consent to participate.

The first round of testing took place at a local school that serves as a center for educational innovation and research. Prior to recruiting any participants, members of the research team met with the school administrators to provide them with details of the study and a demonstration of the prototype. After this initial meeting, the principal spoke with the sixth-grade teachers and determined that a math class would be the best option to participate in the study. Consent forms were sent home to parents. Those who agreed to allow their children to participate were asked to return a signed copy of the consent form to the school. Members of the research team collected consent forms from the school and then worked with the principal and math teacher to schedule a date and time to conduct the test. A total of 22 students returned signed consent forms and were eligible to participate in round 1 of pilot-testing.

The second round of testing took place when many of the local schools were on spring break. In an effort to not retest the same participants, local middle school students were recruited via word of mouth and flyers. In total, 10 parents returned signed consent forms, and their children were eligible to participate in round 2 of pilot-testing. After receiving signed consent forms, research team members worked with parents to schedule a testing day, which took place at a university laboratory space.

During both rounds of pilot-testing, members of the research team explained to the students, in an age-appropriate manner, what participation in the study would entail. If students were interested in participating in the study, they were asked to sign an assent form. Students were then provided with a short video tutorial created by the research team that introduced the assignments they would be working on and instructed them how to scroll and click. Members of the research team asked a few questions to ensure that the students knew how to complete the proper movements. A demonstration was provided if needed. Research team members then fitted the students with accelerometers and a Meta Quest 3 headset. Research team members recorded start and stop times. Additionally, the research team kept note of any exceptional factors that could impact testing (ie, motion sickness or ill-fitting headsets).

### Prototype Program

**Figure 2.**
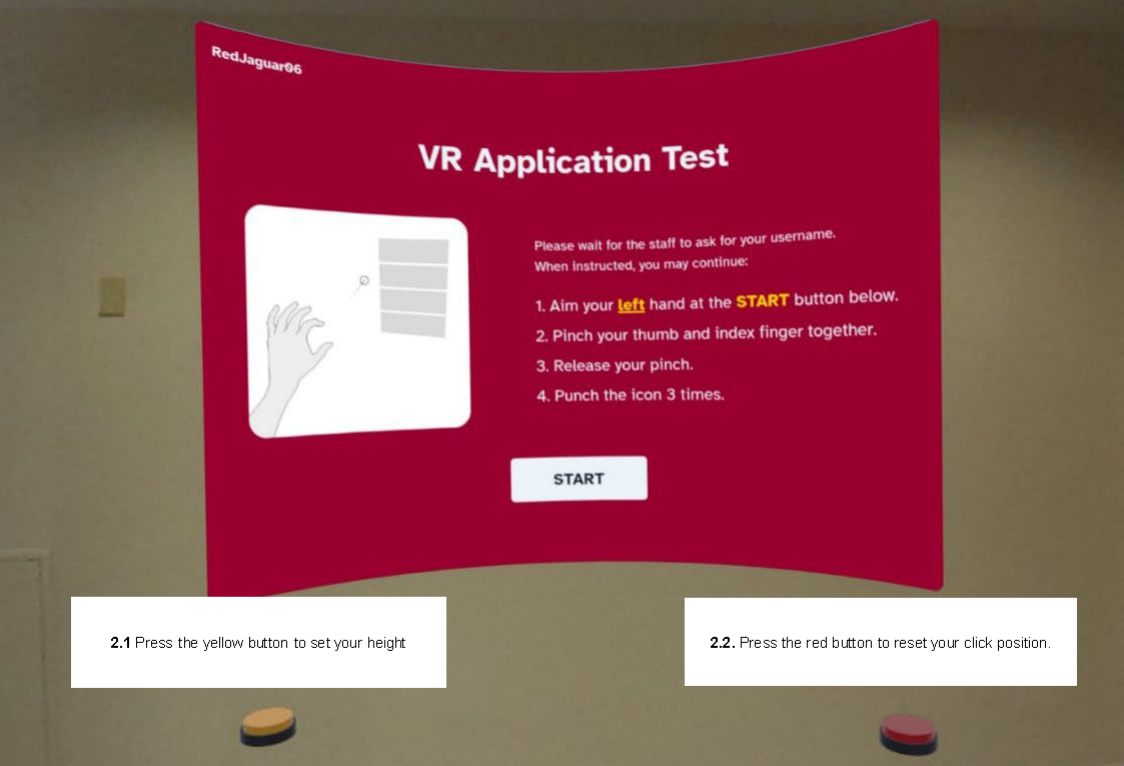
Orientation screen that students viewed when they initially put on the Meta Quest headset, including instructions on how to click, set their default height, and reset the clicker position.

**Figure 3.**
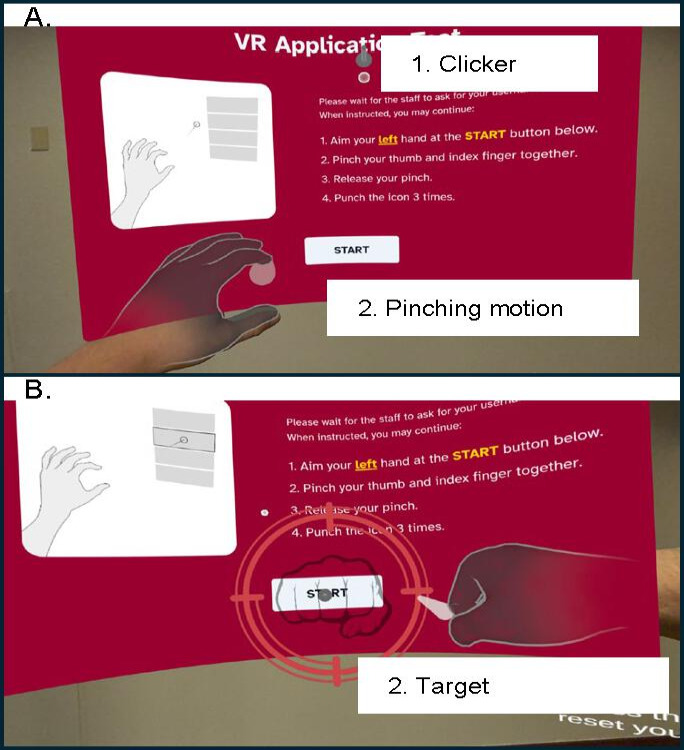
Visual representation of clicking procedures: (1) locating the clicker, (2) making a pinching motion with the thumb and index fingers, and (3) punching the target 3 times. (A) Process to lock in the clicker and (B) process to finalize the click.

A modified virtual reality web browser was used for the online environment during pilot-testing. Upon putting on the Meta Quest 3 headset, students were presented with a large web page (Figure 2) with a yellow button to the left of the screen and a red button to the right. To begin the assignment, students had to press the yellow button, which set their default height. The default height value was used to detect when students jumped or squatted. Once the default height was established, participants could scroll down by jumping and scroll up by squatting. Clicking was a multistep process. Students first aimed with their left hand, which would move a clicker over the screen (Figure 3A). Once the clicker was hovering over the link or button, the participant would make a pinching motion (index finger and thumb closing) to lock the clicker in place. If the student accidentally placed the clicker in the wrong spot, they could press the red button on the right side of the screen to reset it. Once the clicker was locked, a large target appeared (Figure 3B); participants were encouraged to punch the target 3 times to finalize the click. Students were instructed to make hard, fast punches. However, interactions were triggered through collision, not speed. Thus, students could trigger the click with slower punches. Despite these activity-focused interactions, the virtual reality browser otherwise functioned as a normal web browser. It could display arbitrary web content, but for the purpose of this study, the browser was restricted to only showing the standardized academic assignments and the SUS.

### Academic Assignments

During both rounds, students were required to complete academic assignments. For round 1, they completed a vocabulary flash card exercise matching 30 terms to their meaning. If terms were connected to the incorrect meaning, students were required to try again. In addition to the vocabulary assignment, students completed 5 reading comprehension prompts, answering questions related to each prompt. In round 2, they completed the same 2 exercises, but the vocabulary exercise was changed from a flash card format, which did not require scrolling, to a web questionnaire format, which did require scrolling. The updated vocabulary quiz only consisted of 15 questions and did not require students to correct mistakes. This change was made to encourage more physical activity and de-emphasize the notion that students needed to select a “correct” answer, which was not the purpose of this iterative pilot study. In both rounds, the order of these 2 exercises was randomized across participants.

### Measures

#### Physical Activity

Physical activity was assessed using an ActiGraph wGT3X-BT accelerometer (Ametris) worn on a manufacturer-provided band and placed on the right hip in line with the midaxillary line. Research team members helped students attach the accelerometer before being fitted with the headset. Accelerometry wear time was adjusted using recorded headset start and stop times. Accelerometry data were downloaded and analyzed in ActiLife 6 (version 6.15.0; Ametris). Evenson cut points [25], which are recommended for this age group [26], in 15-second epochs were used to categorize physical activity into light, moderate, and vigorous. Percentages of time spent in total physical activity (light, moderate, and vigorous) were used as the outcome variable.

#### Acceptability

The SUS [23,24], which is commonly used to judge the user-friendliness of software systems, was used to measure acceptability. Students answered the 11-item survey in the modified virtual reality web browser. Questions 1 through 10 used a 5-point Likert scale ranging from 1 (“strongly disagree”) to 5 (“strongly agree”). Raw scores are converted to a score of 0 to 100. Lower scores indicated lower usability: scores from 0 to 49.9 correspond to “not acceptable,” scores from 50 to 69.9 correspond to “marginally acceptable,” and scores from 70 to 100 correspond to “acceptable.” In 2009 [24], an adjective rating scale was added to the SUS (question 11). This 11th question aimed to better understand the overall user-friendliness of the prototype using a Likert scale ranging from 1 ( “worst imaginable”) to 7 (“best imaginable”). The 11th item is interpreted on its own and is not included in the total SUS score. Overall, the instrument demonstrates excellent reliability (Cronbach α=0.91) and a high level of concurrent validity (*r*=0.81) [27].

#### Prototype Program

To increase data integrity, data on device use were automatically recorded by the virtual web browser. Specifically, start times, end times, responses to the standardized assignments, SUS scores, and user identifiers were automatically recorded and stored in a remote database. Deidentified assigned nicknames were used to cross-reference physical activity data with performance data.

#### Semistructured Interview

Following completion of their session, students were asked multiple questions that probed their opinion on the experience with the headset program, what they liked most about the experience, and suggestions for improvement.

### Statistical Analysis

The statistical software SPSS (version 29; IBM Corp) was used. Descriptive statistics were calculated for all variables. Mann-Whitney *U* tests were used to identify differences in SUS scores (ie, boys vs girls and rounds 1 vs 2). The α level was set at .05 a priori.

### Ethical Considerations

This study was approved by the institutional review boards of the Pennington Biomedical Research Center (2024-052; approved on October 3, 2024) and Louisiana State University (IRBAM-24-1094; approved on December 5, 2024) and conformed to the latest Declaration of Helsinki. Written parental consent and child assent were obtained before data collection. To maintain privacy and confidentiality, students were assigned a study-specific identification number and a randomly generated code to connect physical activity data with headset use. Students were provided with small tokens of appreciation (ie, notebooks, small fidget toys, stickers, and lip balm) and snacks (ie, fruit, water, granola bars, and fruit snacks) as compensation for their participation.

## Results

### Overview

Results from round 1 and 2 of pilot-testing are presented separately to highlight the changes that occurred during this iterative development process. Demographic characteristics of users in each round are reported in Table 1.

**Table 1.** Characteristics of round 1 and 2 of pilot-testing for the mixed reality prototype.

Variable	Values
Round 1 (n=22)	
Age (years), mean (SD)	12.3 (0.4)
Height (cm), mean (SD)	155.3 (7.6)
Weight (kg), mean (SD)	49.9 (14.5)
BMI (kg/m^2^), mean (SD)	20.2 (4.2)
Healthy weight, n (%)	15 (68.2)
Boys, n (%)	15 (68.2)
White, n (%)	14 (63.6)
Hispanic origin, n (%)	3 (13.6)
Round 2 (n=10)	
Age (years), mean (SD)	11.8 (0.6)
Height (cm), mean (SD)	149.4 (6.5)
BMI (kg/m^2^), mean (SD)	18.6 (3.3)
Healthy weight, n (%)	7 (70.0)
Weight (kg), mean (SD)	41.9 (9.8)
Boys, n (%)	6 (60.0)
White, n (%)	8 (80.0)
Hispanic origin, n (%)	0 (0.0)

### Round 1

**Figure 4.**
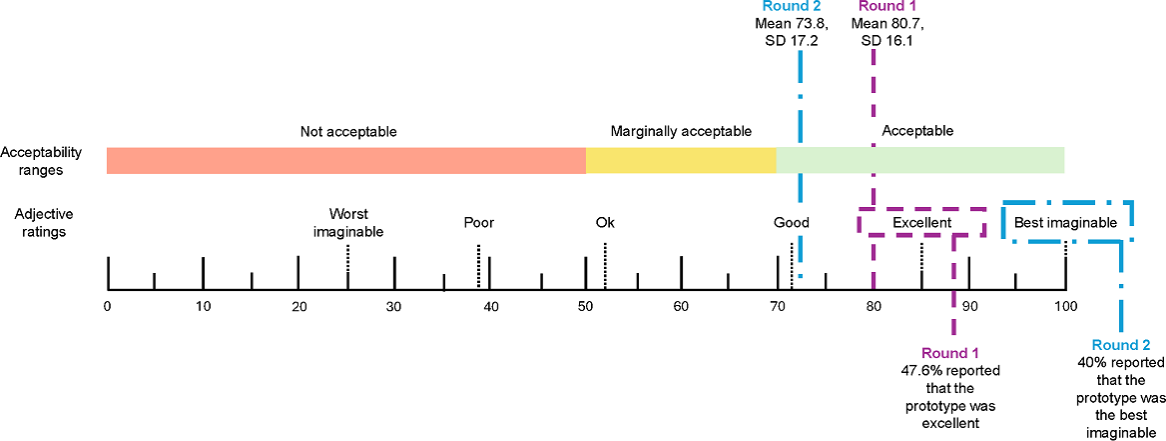
System Usability Scale scores from rounds 1 and 2 of pilot-testing, including acceptability ranges, adjective ratings, and mean and SD of acceptability scores (modified figure used with permission [23,24]).

A total of 22 middle school students (mean age 12.3, SD 0.4 years; n=15, 68.2% boys) participated in round 1 of pilot-testing. Students spent 6.9 (SD 2.7) minutes (46.0%) of the session, which lasted a mean 15 (SD 0) minutes, engaging in physical activity, as shown in Table 2. The average acceptability score was 80.7 (SD 16.1), which was in the highest range for acceptability. Figure 4 [23,24], used with permission, shows how SUS scores are usually interpreted. The average acceptability score of 80.7 lies in the “excellent” range. There were no significant differences (*P*=.79) in acceptability scores between boys (mean 83.17, SD 11.47) and girls (mean 74.58, SD 24.67).

**Table 2.** Physical activity measured through accelerometry during rounds 1 and 2 of pilot-testing for the mixed reality prototype.

Variable	Mean (SD)
Round 1	
Sedentary time (min)	8.1 (2.2)
Light physical activity (min)	4.7 (1.2)
Moderate physical activity (min)	1.1 (0.6)
Vigorous physical activity (min)	1.1 (0.9)
Round 2	
Sedentary time (min)	3.5 (2.0)
Light physical activity (min)	4.2 (2.4)
Moderate physical activity (min)	1.1 (0.3)
Vigorous physical activity (min)	0.5 (0.4)

Semistructured interviews and direct observation revealed several key insights. As suggested by the high SUS score, students indicated that they would be willing to use the prototype to complete school assignments both at home and at school. Students enjoyed most of the features (eg, punching, jumping, sound effects, and ease of use), but some design aspects could be improved. For example, the cursor (pinching and aiming) required precise aim to use; even a small movement could cause a student to miss a button or link. Students suggested making a larger pointer or expanding the selection area to fix this issue. Proctors noticed that some students, instead of punching the target, slapped the target 3 times by making a small movement with their hand.

### Round 2

Following round 1 of pilot-testing, the research team made several adjustments to the prototype to increase physical activity and improve usability. First, the mechanism to scroll was modified to encourage more movement to meet the physical activity goal. In round 1, participants held a squat to scroll the page up. This was modified in round 2 so that, for every squat, the modified web browser would only scroll up a fixed distance, thereby requiring participants to squat repeatedly to scroll up. Second, to require full arm movement, the software was modified to require students’ hands to return to their body after punching before their next punch. Finally, an internal clock was added to allow researchers to precisely analyze physical activity data. In addition to these modifications, researchers changed the vocabulary flash cards to a vocabulary quiz that would require students to scroll the page more. The reading comprehension questions did not change in round 2.

Round 2 participants comprised 10 local middle school students (mean 11.82, SD 0.63 years; n=6, 60% boys). Students were physically active for 5.8 (SD 3.1) minutes (62.4%) of the session, which lasted a mean 9.3 (SD 2.41) minutes (Table 2). The average acceptability score for round 2 was 73.8 (SD 17.2; Figure 4), which remained in the highest category for acceptability and was not statistically significantly different from that in round 1 (*P*=.16). There was no significant difference (*P*=.61) in round 2 acceptability scores between boys (mean 78.75, SD 8.48) and girls (mean 66.25, SD 25.37). Nearly half (4/10, 40%) of the participants reported the user-friendliness of the prototype program as “best imaginable,” which was the highest score. Similarly to round 1 of pilot-testing, the students stated during the semistructured interview that they would use the prototype to complete school assignments. However, a few students stated that, due to the weight of the headset, they would only use the prototype to complete short (15-20 minutes) assignments. Jumping and punching remained the favorite features of both boys and girls. Additionally, the issues with aiming the cursor remained a common complaint.

## Discussion

### Principal Findings

This iterative study resulted in an acceptable mixed reality prototype program that engaged students in physical activity while they completed online academic assignments. To our knowledge, this is the first study to test mixed reality technology as a method for increasing physical activity while completing schoolwork. During round 2 of pilot-testing, students were physically active for most of the time it took them to complete the online assignments, and the students also provided high scores for user satisfaction. Semistructured interviews with students further emphasized that the mixed reality prototype program was a digital learning tool that they would be willing to use at school and at home. Of note, teachers and school administrators were also interested in deploying the mixed reality prototype program in their classes. By conducting 2 rounds of testing with minor modification in between, researchers were able to demonstrate that this prototype can engage students in physical activity 46% to 62.4% of the time it takes to complete online assignments. This ultimately equates to approximately 6 to 7 minutes of physical activity in a 10- to 15-minute academic task. Overall, the results of this study are congruent with those of popular virtual reality exergames such as Beat Saber or Gorilla Tag [21].

### Comparison With Prior Work

Traditionally, school-based physical activity interventions have been designed to incorporate moderate to vigorous physical activity into classroom settings. These methods often result in modest changes in moderate to vigorous physical activity during intervention lessons [28]. Unfortunately, teachers and students might be hesitant to engage in moderate to vigorous physical activity in classroom settings. Teachers often have a limited time to incorporate physical activity into lessons [29], and the classroom size might make it difficult to engage in higher-intensity physical activity. Qualitative data indicate that students are hesitant to engage in higher-intensity physical activity because they do not want to sweat or change clothes [30], and teachers are reluctant due to limited space [31] and competing priorities [32].

Instead, replacing sedentary computer time that is already part of classroom instruction with physical activity computer time may be a less disruptive and more feasible strategy to increase minutes of physical activity. In this study, the developed prototype program elicited similar physical activity responses to those elicited by traditional interventions [28], all while working with a digital learning tool that can be easily incorporated into the learning environment.

These results contribute promising evidence to the nascent research that suggests that mixed reality technology can be used to increase physical activity [20]. Most school-based trials to date that have tested mixed reality, augmented reality, or virtual reality have focused on incorporating activity-promoting technology into physical education classes [22], whereas this prototype was designed to be integrated into a traditional academic subject matter classroom with academic assignments such as reading comprehension. One mixed reality trial integrated into physical education focused on improving children’s motivation to be active and their motor skills and physical fitness but did not evaluate minutes spent engaged in physical activity [33]. A trial that did focus on increasing physical activity was conducted by Ahn et al [34] and involved a virtual fitness buddy ecosystem that would allow children aged 6 to 11 years to interact with a virtual dog to set and meet self-determined physical activity goals. Results indicated that the ecosystem was effective at significantly increasing light physical activity and decreasing sedentary behavior during an after-school program [34]. Similarly to the work by Ahn et al [34], this study found that using a mixed reality prototype primarily increased light physical activity. The integration of movement, even light physical activity, has been shown to benefit physical health and academic outcomes [16-18].

The findings of this study indicate that mixed reality technology might encourage more physical activity during a traditionally sedentary school day. The broader evidence base for exergaming and active video games is mixed. Many have found that these methods are effective at promoting physical activity and decreasing sedentary behavior [20,33-35]. Furthermore, there is evidence suggesting that exergaming and active video games might improve sleep hygiene and health-related quality of life [36]. However, one small trial of 49 children found significant increases in sedentary behavior but no significant increases in other screen time [37]. The overall quality of evidence in active video game research is low, and researchers should aim for increasing methodological rigor and more balanced reporting of findings [38].

### Limitations

This study was designed to examine physical activity while wearing the headset; thus, the difference between typical student classroom activity and activity using our prototype is not known. Although typical in-class assignments are generally sedentary, future trials should examine students’ physical activity levels with and without the prototype, such as comparing physical activity while using a traditional computer vs the mixed reality headset. Additionally, researchers should examine whether integration of the prototype contributes to a meaningful increase in physical activity throughout the school day and whether the effect can be sustained over weeks and months. The assignment time was set for 15 minutes. However, with the addition of an internal clock in round 2, it was evident that students completed the assignment in approximately 9 minutes, and some indicated that the weight of the headset precluded longer sessions. The difference in assignment completion time is most likely attributed to the change in the academic assignment from 30 vocabulary flash cards to a 15-item vocabulary quiz. Future studies should examine the duration of time that students will tolerate wearing the headset while balancing engagement in physical activity and acceptability. Finally, future research should examine acceptability from the classroom teachers and school administrators to ensure that the prototype is helpful and not disruptive to classroom instruction. Anecdotal information from the pilot test indicated that the school administrators were positive about the prototype and the math and science teachers were interested in integrating the mixed reality headset into the classroom, but this should be explored in a structured interview in future trials.

### Conclusions

The continued use of digital learning tools in educational settings has led to increases in screen time and sedentary behavior [7,8,11]. These trends have challenged educators to identify new ways to incorporate movement into classroom settings. The results of this iterative study indicate that this mixed reality prototype program might be a pathway to increase physical activity in the classroom in a way that is acceptable and enjoyed by students. Future research is needed to determine longer-term usability and effectiveness.

## Abbreviations

SUS: System Usability Scale

## References

[1] (2008). Average number of hours in the school day and average number of days in the school year for public schools, by state: 2007–08. U.S. Department of Education, National Center for Education Statistics.

[2] Egan CA, Webster CA, Beets MW (2019). Sedentary time and behavior during school: a systematic review and meta-analysis. Am J Health Educ.

[3] Pearson N, Haycraft E, Johnston JP, Atkin AJ (2017). Sedentary behaviour across the primary-secondary school transition: a systematic review. Prev Med.

[4] (2021). Youth Online - YRBS. Centers for Disease Control and Prevention.

[5] (2002). No Child Left Behind Act of 2001, 115 Stat. 1425. US Government Publishing Office.

[6] (2015). Every Student Succeeds Act, 129 Stat. 1802. US Government Publishing Office.

[7] Siedentop DL (2009). National plan for physical activity: education sector. J Phys Act Health.

[8] Barron AE, Kemker K, Harmes C, Kalaydjian K (2003). Large-scale research study on technology in K–12 schools: technology integration as it relates to the National Technology Standards. J Res Technol Educ.

[9] Delgado A, Wardlow L, O’Malley K, McKnight K (2015). Educational technology: a review of the integration, resources, and effectiveness of technology in K-12 classrooms. J Inf Technol Educ Res.

[10] Pokhrel S, Chhetri R (2021). A literature review on impact of COVID-19 pandemic on teaching and learning. High Educ Future.

[11] Qi J, Yan Y, Yin H (2023). Screen time among school-aged children of aged 6-14: a systematic review. Glob Health Res Policy.

[12] Pfledderer CD, Mullane EJ, Brown DMY (2025). Five-year trends in U. S. child and adolescent 24-hour movement behavior guideline adherence, 2018-2022. J Act Sedentary Sleep Behav.

[13] Şahin F, Doğan E, Okur MR, Şahin YL (2022). Emotional outcomes of e-learning adoption during compulsory online education. Educ Inf Technol (Dordr).

[14] Vachkova SN, Vachkov IV, Klimov IA, Petryaeva EY, Salakhova VB (2022). Lessons of the pandemic for family and school—the challenges and prospects of network education. Sustainability.

[15] Contardo Ayala AM, Parker K, Mazzoli E (2024). Effectiveness of intervention strategies to increase adolescents' physical activity and reduce sedentary time in secondary school settings, including factors related to implementation: a systematic review and meta-analysis. Sports Med Open.

[16] Carson V, Ridgers ND, Howard BJ (2013). Light-intensity physical activity and cardiometabolic biomarkers in US adolescents. PLoS One.

[17] Telford DM, Meiring RM, Gusso S (2023). Moving beyond moderate-to-vigorous physical activity: the role of light physical activity during adolescence. Front Sports Act Living.

[18] Chim HQ, Gijselaers HJM, de Groot RHM, Van Gerven PWM, oude Egbrink MGA, Savelberg H (2024). The effects of light physical activity on learning in adolescents: a systematic review. Int Rev Sport Exerc Psychol.

[19] Buckingham G (2021). Hand tracking for immersive virtual reality: opportunities and challenges. Front Virtual Real.

[20] Odenigbo IP, Reen JK, Eneze C, Friday A, Orji R Virtual, augmented, and mixed reality interventions for physical activity: a systematic review.

[21] Boots B, Berg D, Hewitt E, Naugle K, Naugle K (2025). Physical activity and enjoyment in active virtual reality games in youth: comparative analysis of Gorilla Tag and Beat Saber. JMIR Serious Games.

[22] Pérez-Muñoz S, Castaño Calle R, Morales Campo PT, Rodríguez-Cayetano A (2024). A systematic review of the use and effect of virtual reality, augmented reality and mixed reality in physical education. Information.

[23] Bangor A, Kortum PT, Miller JT (2008). An empirical evaluation of the System Usability Scale. Int J Hum Comput Interact.

[24] Bangor A, Kortum P, Miller J (2009). Determining what individual SUS scores mean: adding an adjective rating scale. J Usability Stud.

[25] Evenson KR, Catellier DJ, Gill K, Ondrak KS, McMurray RG (2008). Calibration of two objective measures of physical activity for children. J Sports Sci.

[26] Trost SG, Loprinzi PD, Moore R, Pfeiffer KA (2011). Comparison of accelerometer cut points for predicting activity intensity in youth. Med Sci Sports Exerc.

[27] Lewis JR (2018). The System Usability Scale: past, present, and future. Int J Hum Comput Interact.

[28] Watson A, Timperio A, Brown H, Best K, Hesketh KD (2017). Effect of classroom-based physical activity interventions on academic and physical activity outcomes: a systematic review and meta-analysis. Int J Behav Nutr Phys Act.

[29] Webster CA, Zarrett N, Cook BS, Egan C, Nesbitt D, Weaver RG (2017). Movement integration in elementary classrooms: teacher perceptions and implications for program planning. Eval Program Plann.

[30] Phillips SR, Marttinen R, Mercier K, Gibbone A (2019). Middle school students’ perceptions of physical education: a qualitative look. J Teach Phys Educ.

[31] Dwyer JJM, Allison KR, Barrera M, Hansen B, Goldenberg E, Boutilier MA (2003). Teachers’ perspective on barriers to implementing physical activity curriculum guidelines for school children in Toronto. Can J Public Health.

[32] Craig DW, Walker TJ, Cuccaro P (2024). Using the R = MC^2^ heuristic to understand barriers to and facilitators of implementing school-based physical activity opportunities: a qualitative study. BMC Public Health.

[33] Bores-García D, Cano-de-la-Cuerda R, Espada M (2024). Educational research on the use of virtual reality combined with a practice teaching style in physical education: a qualitative study from the perspective of researchers. Educ Sci.

[34] Ahn SJG, Schmidt MD, Tate AD (2024). Virtual fitness buddy ecosystem: a mixed reality precision health physical activity intervention for children. NPJ Digit Med.

[35] Staiano AE, Beyl RA, Guan W, Hendrick CA, Hsia DS, Newton RL Jr (2018). Home-based exergaming among children with overweight and obesity: a randomized clinical trial. Pediatr Obes.

[36] Bowling A, Button A, Beyl R (2025). Improving health behaviors and symptoms in youth with mental health disorders: the GamerFit RCT. Ment Health Phys Act.

[37] Ufholz KE, Flack KD, Roemmich JN (2022). The influence of active video game play upon physical activity and screen-based activities in sedentary children. PLoS One.

[38] Moller AC, Sousa CV, Lee KJ, Alon D, Lu AS (2023). Active video game interventions targeting physical activity behaviors: systematic review and meta-analysis. J Med Internet Res.

